# From Behavioral Facilitation to Inhibition: The Neuronal Correlates of the Orienting and Reorienting of Auditory Attention

**DOI:** 10.3389/fnhum.2017.00293

**Published:** 2017-06-06

**Authors:** Faith M. Hanlon, Andrew B. Dodd, Josef M. Ling, Juan R. Bustillo, Christopher C. Abbott, Andrew R. Mayer

**Affiliations:** ^1^The Mind Research Network/Lovelace Biomedical and Environmental Research Institute, AlbuquerqueNM, United States; ^2^Department of Psychiatry, University of New Mexico School of Medicine, AlbuquerqueNM, United States; ^3^Department of Neurosciences, University of New Mexico School of Medicine, AlbuquerqueNM, United States; ^4^Department of Neurology, University of New Mexico School of Medicine, AlbuquerqueNM, United States; ^5^Department of Psychology, University of New Mexico, AlbuquerqueNM, United States

**Keywords:** exogenous attention, inhibition of return, ventral frontoparietal, dorsal frontoparietal, fMRI, ventrolateral prefrontal cortex, stimulus onset asynchrony (SOA)

## Abstract

Successful adaptive behavior relies on the ability to automatically (bottom-up) orient attention to different locations in the environment. This results in a biphasic pattern in which reaction times (RT) are faster for stimuli that occur in the same spatial location (valid) for the first few hundred milliseconds, which is termed facilitation. This is followed by faster RT for stimuli that appear in novel locations (invalid) after longer delays, termed inhibition of return. The neuronal areas and networks involved in the transition between states of facilitation and inhibition remain poorly understood, especially for auditory stimuli. Functional magnetic resonance imaging (fMRI) data were therefore collected in a large sample of healthy volunteers (*N* = 52) at four separate auditory stimulus onset asynchronies (SOAs; 200, 400, 600, and 800 ms). Behavioral results indicated that facilitation (valid RT < invalid RT) occurred at the 200 ms SOA, with inhibition of return (valid RT > invalid RT) present at the three longer SOAs. fMRI results showed several brain areas varying their activation as a function of SOA, including bilateral superior temporal gyrus, anterior thalamus, cuneus, dorsal anterior cingulate gyrus, and right ventrolateral prefrontal cortex (VLPFC)/anterior insula. Right VLPFC was active during a behavioral state of facilitation, and its activation (invalid – valid trials) further correlated with behavioral reorienting at the 200 ms delay. These results suggest that right VLPFC plays a critical role when auditory attention must be quickly deployed or redeployed, demanding heightened cognitive and inhibitory control. In contrast to previous work, the ventral and dorsal frontoparietal attention networks were both active during valid and invalid trials across SOAs. These results suggest that the dorsal and ventral networks may not be as specialized during bottom-up auditory orienting as has been previously reported during visual orienting.

## Introduction

Successful adaptive behavior relies on the ability to automatically (i.e., bottom-up) orient attention to different locations in the environment based on unknown incoming sensory information. These involuntary, bottom-up driven shifts of attention are also called exogenous orienting (Jonides and Irwin, 1981; Mondor and Bryden, 1992; Spence and Driver, 1994; Mondor and Breau, 1999). During exogenous orienting, attention is initially directed to the general location of a sensory cue, resulting in faster reaction times (RT) for targets occurring at the cued location vs. elsewhere in the peripheral environment (i.e., facilitation; Posner et al., 1982; Posner and Cohen, 1984; Posner, 2014). However, it is more ecologically advantageous for organisms to redirect their attention to novel locations after a short period of time (Mondor et al., 1998; Wang and Klein, 2010), resulting in slower RT to cued locations (i.e., inhibition of return; IOR). Although the behavioral and electrophysiological outcomes associated with facilitation and IOR have been extensively studied (Chica et al., 2014b; Martin-Arevalo et al., 2015), the neuronal areas and networks involved in the transition between the two states remain relatively unknown, especially in the auditory modality.

Exogenous orienting is usually induced by presenting a peripheral cue (e.g., tone pip) that predicts an upcoming target location at chance levels: 50% of the cues predict the location correctly, presented at the same hemifield (i.e., valid trials) and 50% of the cues predict the location incorrectly, presented at a different hemifield (i.e., invalid trials). This produces a biphasic response pattern, with faster RT for valid than invalid trials at short (100–250 ms) stimulus onset asynchronies (SOAs), followed by faster RTs for invalid than valid (IOR) trials at longer (400–3000 ms) SOAs (Spence and Driver, 1998; Mondor, 1999; Mondor and Breau, 1999; Tassinari et al., 2002). These exogenous orienting mechanisms are thought to be initiated by salient events, and stimulus-driven.

A predominant theory suggests that there are two neuronal networks that mediate the orienting response (Corbetta and Shulman, 2002; Vossel et al., 2014). The dorsal frontoparietal network, including intraparietal sulcus, superior parietal lobule, and frontal eye fields, responds when attention is voluntarily oriented (i.e., top-down or endogenous orienting) to stimuli in space following valid trials, providing a direct link between the sensory stimuli and appropriate motor responses. In contrast, the ventral frontoparietal network, including the temporoparietal junction, ventrolateral prefrontal cortex (VLPFC; which includes the inferior frontal gyrus), anterior insula and middle frontal gyrus, is activated when stimuli occur unexpectedly or outside the focus of attention (i.e., bottom-up or exogenous orienting). Although the ventral and dorsal systems are thought to be specialized in function, both are activated during visual reorienting due to the multiple requirements that occur following an invalid cue (Kincade et al., 2005; Corbetta and Shulman, 2011). Similarly, IOR is also thought to be mediated by regions from both the ventral and dorsal attention networks (Chica et al., 2011, 2014a). Although distinctions between the two networks have primarily been studied in the visual modality (Chica et al., 2013), it has been suggested that they respond in a supramodal manner (Macaluso, 2010).

However, there is some evidence that the networks mediating auditory orienting may differ from those in the visual modality (Mayer et al., 2006, 2007, 2009; Salmi et al., 2009). For example, Salmi et al. (2009) examined brain activation differences between top-down controlled (visual cue) and bottom-up triggered attention to auditory targets. They found both types of attention activated a widespread overlapping network that included areas from the ventral (VLPFC, middle frontal gyrus, and temporoparietal junction) and dorsal (frontal eye field/premotor cortex and superior parietal lobule) frontoparietal networks. Similarly, Mayer et al. (2007, 2009) and Teshiba et al. (2013) have found bottom-up exogenous reorienting to activate a large-scale frontoparietal-cerebellar network for both facilitation and IOR. Specifically they found activation in pre-SMA/SMA, cingulate, superior, middle, and inferior frontal gyrus, insula, precuneus/superior and inferior parietal lobe, and cerebellum during the disengagement of attention (invalid > valid) at short SOAs (Mayer et al., 2007, 2009; Teshiba et al., 2013), followed by a reversal of activity (valid > invalid) for several of these structures during auditory IOR (800 ms; Mayer et al., 2007, 2009). Visual and auditory orienting may also differ on their reliance of the ventral and dorsal attention networks due to the diverse underlying neuronal mechanisms related to basic physiology (i.e., the spatial advantage of visual information due to the direct mapping of the retina on the visual cortex; Witten and Knudsen, 2005) or basic psychophysics (i.e., the adaptation of a centrally presented arrow cue to direct visual spatial attention relative to complex, spectrally varying tones to shift auditory spatial attention; Mayer et al., 2009). Moreover, the dorsal network also includes regions (inferior and superior parietal cortices) commonly implicated in the auditory “where” stream whereas the ventral network includes areas (VLPFC) implicated in the auditory “what” stream (Wang et al., 2008), suggesting that task requirements may also affect network activation during auditory orienting.

Thus, both attention networks are active during auditory exogenous reorienting. However, it is still unclear how areas within the ventral and dorsal networks participate in the behavioral transition from exogenous facilitation (RT valid < invalid trials) to IOR (RT invalid < valid). Areas which may potentially be involved in this transition between the two states include the middle frontal gyrus and inferior frontal gyrus. Patients with lesions to these frontal regions have been found to show more facilitation at short SOAs and to continue showing facilitation at long SOAs, when healthy controls are exhibiting IOR (Snyder and Chatterjee, 2006). Thus, these frontal areas could possibly be involved in the reorienting of their attention from the valid targets during facilitation to the invalid targets during IOR.

The present study used event-related functional magnetic resonance imaging (fMRI) to investigate the neural networks underlying behavioral exogenous auditory facilitation (200 ms SOA) and IOR (800 ms SOA) in a large sample of healthy volunteers. Two other SOAs (400 and 600 ms) were included to fully map the transition between these two attentional states. We predicted that both the ventral and dorsal system would be activated following attentional reorienting at the shortest SOA (to invalid location at 200 ms) and during IOR (to valid location at 800 ms; Validity × SOA interaction). However, the direction of activation between valid and invalid trials should switch to mirror behavioral data as reported in previous auditory exogenous orienting studies (Mayer et al., 2007, 2009). Finally, the validity index (invalid – valid trials) was also used to more directly map the relationship between behavior and functional activation within areas of the dorsal and ventral networks. We predicted that this analysis would be more sensitive given the known individual differences in how participants use information provided by non-informative cues (see Mayer et al., 2007 for individual variability).

## Materials and Methods

### Participants

Fifty-seven healthy adult volunteers were recruited to participate in the current study through community advertisements. Data from a subset of the current cohort were previously published in a study examining orienting in patients with schizophrenia (Abbott et al., 2012). One participant was identified as a motion outlier (more than three times the interquartile range on 2 of 6 framewise displacement parameters) and was removed from subsequent analyses. Several additional participants were removed for poor behavioral performance during the task [RT outlier (*N* = 1) or accuracy below 70% on any single trial type (*N* = 3)]. The final cohort included 52 participants (40 males; mean age = 34.21 ± 11.91 years old).

Participants were excluded if they had any current or past Axis I disorder as assessed with the Structured Clinical Interview for the Diagnostic and Statistical Manual of Mental Disorders-IV, Research Version, Non-Patient Edition (First et al., 2002). Inclusion criterion were the following: (1) no current or past diagnosis of neurological disorder, history of head trauma (loss of consciousness > 5 min), or mental retardation; (2) no diagnosis of active substance dependence or abuse within the last 12 months (except for nicotine) and no past dependence on or any use in the past 12 months of PCP/Amphetamine/Cocaine; and (3) 18–65 years of age. The University of New Mexico Human Research Review Committee approved this study and all participants provided written informed consent prior to study enrollment.

### Experimental Design and Task

**FIGURE 1:**
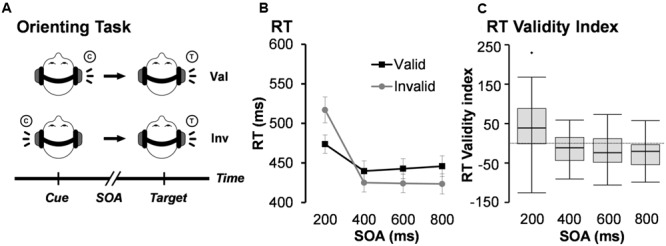
**(A)** Presents a diagrammatic representation of the exogenous auditory orienting task. Headphones were used to present a 2000 Hz pure tone [the cue (C)], which either correctly (valid trials; Val) or incorrectly (invalid; Inv) predicted the location of a second 1000 Hz tone [the target (T)] on 50% of trials. The cue and target were separated with a stimulus onset asynchrony (SOA) of either 200, 400, 600, or 800 ms. Participants indicated the spatial location of the target by pressing a key with their right index (left target) or right middle (right target) finger. **(B)** Depicts average median reaction time (RT) for valid and invalid trials at each of the four different SOAs. Error bars represent standard error of the mean. **(C)** Presents box-and-whisker plots of the validity index (RT invalid – RT valid trials) for each SOA.

Participants completed practice trials of the exogenous auditory orienting task (**Figure 1A**) prior to performing this task in a 3T Siemens TrioTim scanner. Auditory stimuli were presented via an Avotec Silent Scan 3100 Series System using Presentation software (Neurobehavioral Systems). Visual stimuli consisted of a white fixation cross (visual angle = 1.02°) on a black background that was rear projected onto an opaque white Plexiglas projection screen. Participants were instructed to keep their eyes fixated on the cross during the task.

Auditory cues (100 ms, 2000 Hz tone pip) were presented to the left or right ear and correctly (i.e., valid trials) predicted the location of the targets (100 ms, 1000 Hz tone pip) on 50% of the experimental trials. Cues and targets were presented at the same intensity level for all participants and included 10 ms linear onset-offset ramps to minimize clicks. Participants were specifically informed that cues would not contain useful information about the location of the target to maximize exogenous orienting. Participants were instructed to press a button with their right middle finger for target tones presented to the right headphone and a button with their right index finger for target tones presented to their left headphone. Short (200 ms) and long (800 ms) SOAs, respectively, captured facilitation and IOR, with two additional SOAs (400 and 600 ms) capturing the transition between orienting states. Trials were pseudorandomly presented based on both trial-type (Validity) and SOA. The inter-trial intervals (ITI) varied (6 ± 2 s) to decrease temporal expectations and permit modeling of the hemodynamic response function (HRF). Participants completed a total of 224 trials (28 trials for each of the 8 conditions) across four separate runs. Within-subject effect sizes were calculated based on previously published methods by Morris and DeShon (2002). This method corrects for known covariation between paired variables and is comparable in magnitude to Cohen’s d effect sizes.

### Imaging Data Acquisition

Structural images were collected with magnetization-prepared 180° radio-frequency pulses and rapid gradient-echo (MPRAGE) sequence [TEs (echo time) = 1.64, 3.5, 5.36, 7.22, and 9.08 ms; TR (repetition time) = 2.53 s; flip angle = 7°; NEX (number of excitations) = 1; slice thickness = 1 mm; FOV (field of view) = 256 mm; and resolution = 256 × 256]. Functional images were collected with a single-shot, gradient-echo echo-planar pulse sequence [TE = 29 ms; TR = 2000 ms; flip angle = 75°; FOV = 240 mm; voxel size: 3.75 mm × 3.75 mm × 4.55 mm]. The first image of each run was eliminated secondary to T1 equilibrium effects along with two dummy scans leaving a total of 688 images for the final analyses.

### Imaging Data Analysis

First level and group statistics were carried out using the Analysis of Functional Neuroimages (AFNI; Cox, 1996). Time series data were despiked, temporally interpolated to correct for slice time acquisition differences, spatially registered (two and three dimensionally) to the second image of the first run, converted to a standard stereotaxic coordinate space (Talairach and Tournoux, 1988) using a non-linear algorithm, and blurred using a 6 mm Gaussian full-width half-maximum filter. Deconvolution was used to generate a HRF on a voxel-wise basis that spanned the first 16 s post-stimulus onset from the cue for all trials in each condition (valid and invalid trials at each SOA of four SOAs). Six rigid-body motion parameters and their derivatives were included as regressors of no interest to minimize the effect of head motion. Image smoothness was estimated using the residualized timeseries data and spherical autocorrelation (Cox et al., 2017). The third and the fourth images (4.0–8.0 s post-stimulus onset from the cue, corresponding to the peak of the HRF) were averaged and divided by the baseline coefficient to obtain an estimate of percent signal change (PSC). The baseline state consisted of visual fixation and passive exposure to acoustic noise from gradient switching.

A whole-brain, voxel-wise 2 × 4 [Validity (Invalid and Valid) × SOA (200, 400, 600, and 800 ms)] ANOVA was performed on the spatially normalized PSC data using the 3dMVM module in AFNI. The validity index (invalid – valid) was used to directly assess the relationship between behavioral (RT; independent variable) and functional (PSC; dependent variable) data for each SOA (200, 400, 600, and 800 ms) on a voxel-wise basis. All functional results were corrected for false positives at *p* < 0.05 (*p* < 0.001; minimum cluster size = 704 μl) based on 10,000 Monte-Carlo simulations.

## Results

### Behavioral Performance

See **Table 1** for a summary of behavioral performance descriptive statistics. Behavioral accuracy data were non-normally distributed and approached ceiling (>98%) for all trial types. As a result, they were not analyzed further. A 2 × 4 (Validity × SOA) repeated measures ANOVA was performed on RT data for correct trials only to evaluate task performance (**Figures 1B,C**). RT data was normally distributed for valid and invalid trials at the 400, 600, and 800 ms SOAs, but not at the 200 ms SOA. The analysis indicated a significant main effect for SOA (*F*_3,49_ = 68.29, *p* < 0.001), with increased RT to the shortest relative to longer SOAs. The main effect of Validity was not significant (*F*_1,51_ = 0.39, *p* = 0.535). There was a significant Validity × SOA interaction (*F*_3,49_ = 34.24, *p* < 0.001). Follow-up paired-samples *t*-tests at each SOA (Bonferroni corrected *p*-value is 0.05/4 = 0.0125) showed faster RT for valid than invalid trials at 200 ms SOA (*t*_51_ = -4.52, *p* < 0.001), and the opposite pattern (i.e., faster RT for invalid trials) at 600 ms (*t*_51_ = 3.08, *p* = 0.003) and 800 ms (*t*_51_ = 4.87, *p* < 0.001) SOAs. Invalid trials were also faster than valid trials at the 400 ms SOA, but did not survive correction for multiple comparisons (*t*_51_ = 2.58, *p* = 0.013).

**Table 1:** Summary of behavioral performance descriptive statistics.

SOA	200 ms			400 ms			600 ms			800 ms		
Validity	Invalid	Valid	Effect size	Invalid	Valid	Effect size	Invalid	Valid	Effect size	Invalid	Valid	Effect size
Percent	98.01	99.31	–0.30	99.18	99.31	–0.07	99.18	98.90	0.12	99.66	98.63	0.44
Correct	(4.33)	(1.74)		(1.95)	(2.13)		(2.51)	(2.30)		(1.06)	(2.93)	
Reaction	517.05	473.77	0.71	425.04	439.68	–0.36	424.15	442.76	–0.43	423.50	445.87	–0.68
Time	(118.05)	(83.53)		(85.41)	(93.31)		(84.43)	(91.08)		(91.36)	(93.92)	

*Mean and standard deviation in parentheses listed for accuracy (percent correct) and median reaction time during each stimulus onset asynchrony (SOA), as well as effect size for within-subjects comparisons (Morris and DeShon, 2002).*

The magnitude of the validity index (RT invalid – RT valid trials) also indicated a significant effect of SOA (*F*_3,49_ = 34.24, *p* < 0.001; **Figure 1C**). Follow-up paired-samples *t*-tests compared the magnitude of the validity effect at successive SOAs correcting for multiple comparisons (Bonferroni corrected *p*-value is 0.5/3 = 0.017). Results indicated significant differences in the validity effect between 200 (mean ± standard deviation = 43.29 ± 69.13) and 400 (mean ± standard deviation = -14.63 ± 40.87) ms SOAs (*t*_51_ = 6.18, *p* < 0.001; effect size = 0.91). There were no significant differences and small effect sizes between 400 and 600 (mean ± standard deviation = -18.61 ± 43.53) ms SOA trials (*t*_51_ = 0.73, *p* = 0.469; effect size = 0.10), as well as between 600 and 800 (mean ± standard deviation = -22.36 ± 33.12) ms SOA trials (*t*_51_ = 0.78, *p* = 0.442; effect size = 0.11). Collectively, these behavioral results suggest significant reorienting at the 200 ms SOA in conjunction with comparable IOR effects at the 400, 600, and 800 ms SOAs. Considerable variation was observed across individuals in terms of their use of bottom-up cue information.

### Functional Imaging Results

**FIGURE 2:**
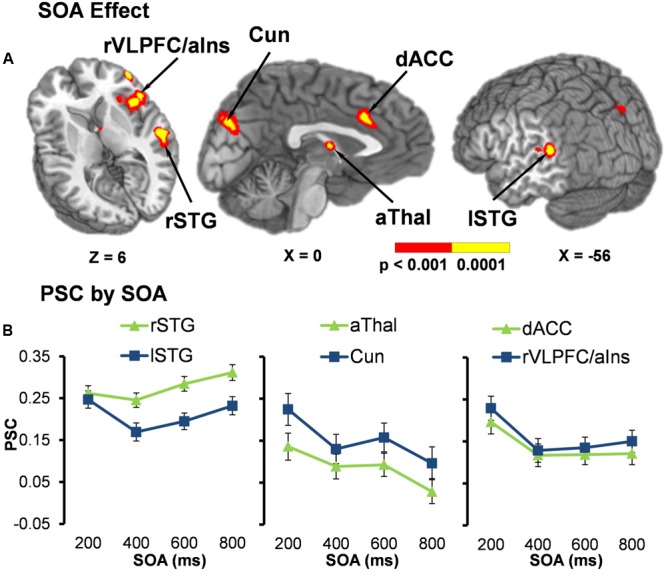
**(A)** Displays the regions of the brain showing a significant main effect of stimulus onset asynchrony (SOA) (*p* < 0.001: red; *p* < 0.0001: yellow). Locations of the sagittal (X) and axial (Z) slices are given according to the Talairach atlas. **(B)** Displays the average percent signal change (PSC) for each significant region and at each SOA (200, 400, 600, and 800 ms), separated into three graphs for each pattern of activation found. The first pattern showed the right (rSTG) and left (lSTG) superior temporal gyrus decreasing in activation from 200 ms to 400 ms SOA trials, followed by increased activation as a function of increasing SOA. Second, activation within the anterior thalamus (aThal) and bilateral cuneus/precuneus (Cun) generally decreased as a function of SOA. Finally, activation in the dorsal anterior cingulate gyrus (dACC) and right ventrolateral prefrontal cortex/anterior insula (rVLPFC/aIns) was at its highest during 200 ms SOA and then remained constant across all other SOAs. Error bars represent the standard error of the mean.

Functional results indicated a significant main effect of SOA across six different regions bilaterally (**Table 2**). Activation was highest for all but one area [right superior temporal gyrus (STG)] at the 200 ms SOA, followed by three general patterns of activation as a function of increasing SOA (**Figure 2** and **Table 3**). The first pattern was observed within the right (BAs 21/22/41/42) and left STG (extending into the inferior parietal lobule; BAs 22/39/40). Activation decreased at 400 ms SOA trials in left STG, followed by increased activation as a function of increasing SOA bilaterally. Second, activation within the anterior thalamus and bilateral cuneus/precuneus (BAs b. 18/19, l. 7/31) generally decreased from 200 to 400 ms SOA trials, and from 600 to 800 ms SOA trials. Finally, activation in the dorsal anterior cingulate gyrus (dACC; BAs b. 32, l. 24) and right inferior frontal gyrus (VLPFC)/anterior insula (BAs 13/22/44/45/46/47) was at its highest during 200 ms SOA, dropping significantly at 400 ms, and then remained constant across all other SOAs. No areas were significant for the main effect of Validity.

**Table 2:** Summary of significant clusters for fMRI results.

	Cluster volume (μl)	Center of mass *X, Y, Z*	*X* range	*Y* range	*Z* range	Maximum intensity *X, Y, Z*
**SOA main effect**						
*rSTG*	3174	57.6, -32.0, 9.1	49 to 67	–51 to -19	–7 to 19	61, -26, 9
*lSTG*	1428	–57.8, -46.0, 17.0	–64 to -51	–56 to -35	8 to 34	–56, -51, 14
*aThal*	788	4.2, -3.6, 11.4	–4 to 13	–9 to 1	6 to 20	3, -4, 10
*Cun*	1756	–0.2, -78.4, 33.2	–8 to 8	–87 to -72	22 to 41	–2, -81, 35
*dACC*	826	0.7, 22.8, 28.6	–3 to 4	16 to 30	21 to 36	1, 22, 29
*rVLPFC/aIns*	5110	48.6, 17.6, 3.3	31 to 62	3 to 46	–8 to 14	45, 12, 5
**200 ms SOA validity index regression**						
*rVLPFC/aIns*	1360	38.5, 22.4, 0.6	28 to 49	16 to 28	–7 to 5	32, 22, 0

*SOA, stimulus onset asynchrony; rSTG, right superior temporal gyrus; lSTG, left superior temporal gyrus; aThal, anterior thalamus; Cun, cuneus/precuneus; dACC, dorsal anterior cingulate gyrus; rVLPFC/aIns, right ventrolateral prefrontal cortex/anterior insula. Coordinates are provided in Talaraich space.*

**Table 3:** Pairwise comparisons of activation between trials at each stimulus onset asynchrony (SOA) for areas with significant main effect of SOA.

	200 vs. 400 ms				400 vs. 600 ms			600 vs. 800 ms		
SOA main effect	200 ms	400 ms	*t*_51_-value	*p*-value	600 ms	*t*_51_-value	*p*-value	800 ms	*t*_51_-value	*p*-value
rSTG	0.26 (0.13)	0.25 (0.12)	1.05	0.299	0.28 (0.13)	–2.04	**0.046**	0.31 (0.14)	–2.80	**0.007**
lSTG	0.25 (0.15)	0.17 (0.16)	3.85	**<0.001**	0.19 (0.14)	–1.21	0.234	0.23 (0.16)	–2.81	**0.007**
aThal	0.14 (0.23)	0.09 (0.21)	1.93	0.059	0.09 (0.20)	–0.18	0.858	0.03 (0.23)	2.95	**0.005**
Cun	0.22 (0.27)	0.13 (0.25)	3.39	**0.001**	0.16 (0.25)	–0.92	0.361	0.10 (0.28)	2.77	**0.008**
dACC	0.20 (0.21)	0.12 (0.19)	4.20	**<0.001**	0.12 (0.17)	–0.06	0.955	0.12 (0.19)	–0.15	0.882
rVLPFC/aIns	0.23 (0.21)	0.13 (0.20)	4.62	**<0.001**	0.14 (0.18)	–0.25	0.802	0.15 (0.19)	–0.96	0.341

*Mean percent signal change and standard deviation in parentheses for each area showing significant main effect across SOA in functional imaging analysis. rSTG, right superior temporal gyrus; lSTG, left superior temporal gyrus; aThal, anterior thalamus; Cun, cuneus/precuneus; dACC, dorsal anterior cingulate gyrus; rVLPFC/aIns, right ventrolateral prefrontal cortex/anterior insula. *P*-values are marked with a less-than sign (<) if they are less than 0.001 and bold text if they are significant.*

**FIGURE 3:**
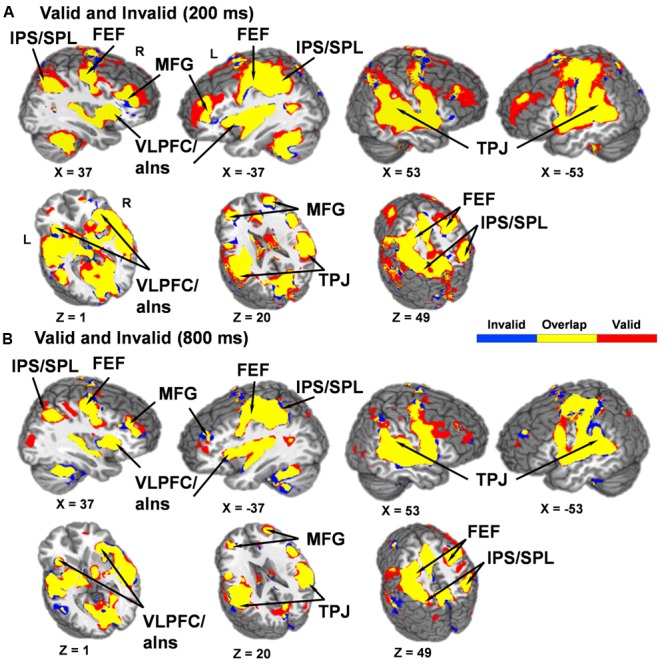
Results from *post hoc t*-tests comparing activation during both valid and invalid trials relative to baseline at the 200 **(A)** and 800 **(B)** ms SOAs. Areas significantly activated for invalid trials are displayed in blue color and areas significantly activated for valid trials are displayed in red color, with the overlap of activation for both invalid and valid trials displayed in yellow (*p* < 0.001). Locations of the sagittal (X) and axial (Z) slices are given according to the Talairach atlas for the left (L) and right (R) hemispheres. A qualitative examination of the functional maps indicates that valid and invalid trials at both SOAs activated key structures within the dorsal [intraparietal sulcus (IPS), superior parietal lobule (SPL), and frontal eye fields (FEF)] and ventral [temporoparietal junction (TPJ), ventrolateral prefrontal cortex/anterior insula (VLPFC/aIns) and the middle frontal gyrus (MFG)] networks.

Contrary to our a priori predications, no areas were significant for the Validity × SOA interaction. Thus, *post hoc* whole-brain, voxel-wise *t*-tests were conducted to determine if the dorsal and ventral attention streams were both activated during orienting (valid trials) and reorienting (invalid trials) relative to baseline at the 200 ms (facilitation) and 800 ms (IOR) SOAs or inactivated across both trial types as either pattern would result in a null interaction effect. Results indicated that the dorsal and ventral attention streams were active during orienting and reorienting trials relative to baseline at both SOAs (**Figure 3**).

**FIGURE 4:**
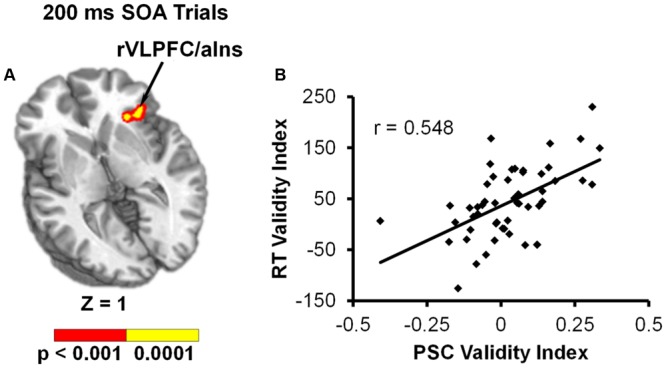
Results from voxel-wise linear regression examining the relationship between RT validity index (invalid – valid) and PSC validity index (invalid – valid) during the 200 ms SOA trials. **(A)** Shows that functional activation in the right ventrolateral prefrontal cortex/anterior insula (rVLPFC/aIns) was significantly related to behavioral reorienting (*p* < 0.001: red; *p* < 0.0001: yellow). Location of the axial (Z) slice is given according to the Talairach atlas. **(B)** Displays a scatterplot of this relationship.

We predicted that the behavioral validity index (invalid RT – valid RT) would serve as a more sensitive measure for capturing differential use of the cues across participants. Results from the voxel-wise linear regressions with RT validity index as the independent variable and PSC validity index as the dependent variable for each SOA indicated that behavior was only significantly associated with functional activity at the 200 ms SOA. Specifically, the RT validity index was positively correlated with the PSC validity index in the right inferior frontal gyrus (VLPFC) and anterior insula (BAs 13/45/47; see **Table 2** and **Figure 4**).

## Discussion

The present study examined how the dorsal and ventral frontoparietal networks mediate the transition between auditory facilitation and IOR. Current behavioral results provide replication of previous research (Mondor et al., 1998; Chica et al., 2014b), with facilitation (valid RT < invalid RT) present at the 200 ms and IOR (valid RT > invalid RT) present at the 600 and 800 ms SOAs. Importantly, these behavioral results suggest that the majority of trials (75%) occurred during a relative state of inhibition. Contrary to *a priori* predictions and previous results (Mayer et al., 2007), activation within the ventral and dorsal system did not vary as a function of cue validity and SOA. Instead, several regions varied only as a function of SOA, whereas the dorsal and ventral frontoparietal networks were equally active during both valid and invalid trials across all SOAs. Finally, an analysis examining the relationship between response time data and the degree of differential brain activation highlighted the role of the right VLPFC/anterior insula during attentional reorienting at the 200 ms SOA.

Current results indicated six brain regions where activation varied across attentional states, dependent on whether facilitation or IOR was observed in the behavioral data. Specifically, activation in the bilateral dACC and right VLPFC/anterior insula were at their highest when facilitation behaviorally occurred (200 ms SOA), and then remained relatively constant across all other SOAs during IOR. Increased activation of these structures is most likely related to the heightened demand for cognitive and inhibitory control when attention must be quickly deployed or redeployed following targets at short SOAs (Pasternak and Greenlee, 2005; Bari and Robbins, 2013). The dACC and VLPFC have been shown to be involved in conflict monitoring, response inhibition and response selection (Braver et al., 2001; Botvinick, 2007; Mayer et al., 2012; Bari and Robbins, 2013), all of which are likely to be greater at short SOAs. In addition, the VLPFC represents a key node of the ventral attention network (Corbetta and Shulman, 2011; Vossel et al., 2014).

The right VLPFC/anterior insula was also the only region to show a direct relationship between functional activation and response time data (i.e., invalid – valid trials), and this finding was only present at the shortest SOA. The VLPFC has been proposed to play a key role in mitigating the interaction between the dorsal and ventral networks (Asplund et al., 2010; Vossel et al., 2014). Resting-state data suggests strong connectivity between the right VLPFC and both the ventral and dorsal networks (Fox et al., 2006; He et al., 2007). Although it is not clear what exact role the right VLPFC is playing during behavioral facilitation, previous meta-analyses (Levy and Wagner, 2011) indicate that different aspects of the right VLPFC may be activated more for motor inhibition (i.e., posterior-VLPFC), response uncertainty (i.e., mid-VLPFC), and exogenous reorienting (i.e., anterior-VLPFC). The right VLPFC activation found in the current study included all of these aspects, along with the anterior insula. This suggests that the right VLPFC may be initiating a variety of functions (i.e., response inhibition, response uncertainty, and reorienting) at the 200 ms SOA.

Activation within the anterior thalamus and bilateral cuneus/precuneus generally decreased as participants transitioned from a state of facilitation to IOR. The thalamus has been implicated in alerting and shifting of attention (Fan et al., 2005), both of which are likely to be greater when a target occurs immediately following a cue. In addition, activation of the cuneus has been found during behaviorally difficult auditory attention tasks that have an unseen sound source (i.e., auditory occipital activation; Cate et al., 2009), which is the case for exogenous reorienting. In contrast, the magnitude of activation within bilateral STG generally decreased from 200 to 400 ms SOA trials, and then increased significantly over the longest SOA trials during IOR. Increased activation of bilateral STG for longer SOAs (i.e., 100 to 800 ms) was also observed in our previous study (Mayer et al., 2007). In this study we discuss that these auditory regions have been associated with an auditory working-memory system (Pasternak and Greenlee, 2005; Rama and Courtney, 2005) and could be activated more at longer SOAs due to the increased working memory demand of maintaining the cued location in a temporary buffer. The increased activation within the STG could also simply be a result of greater summing of the HRFs from longer sustained activation (i.e., maintaining the cued location) as the SOA between the cue and target stimuli increases. Increased activation in the STG could be related to the foreperiod effect (i.e., increased response time as SOA increases), which is seen in our behavioral data and previous studies on orienting (Mayer et al., 2006, 2007).

Alternatively, this increase in STG activation could be due to differences in processing the laterality of stimulus presentation. We have previously reported evidence of asymmetrical auditory cortex activation from facilitation (contralateral model; right STG responding to left stimulus presentation and left STG responding to right stimulus presentation) to IOR (neglect model; right STG responding to bilateral stimulus presentation and left STG to right stimulus presentation) during auditory orienting tasks (Teshiba et al., 2013). Thus, the current finding of increased STG activation during both states of attention, especially right STG during IOR, may reflect the behavioral needs of that state. Facilitation requires focused attention on the location of the auditory cue and target, thus primarily activating the contralateral STG. In contrast, IOR requires attention to shift from the cue to novel locations, including both hemifields, thus activating the right STG regardless of the laterality of the stimulus presentation.

Current principle and supplementary analyses indicated that the ventral and dorsal networks were both activated during valid and invalid trials during behavioral facilitation (200 ms SOA) and IOR (800 ms SOA). In contrast, previous studies have suggested either increased activity for invalid trials within large-scale frontoparietal-cerebellar networks (Mayer et al., 2006, 2007, 2009; Salmi et al., 2007, 2009), as well as functional activation which mirrors behavioral data during both facilitation and IOR (Mayer et al., 2007). The lack of a Validity × SOA interaction in the current study may be due the inclusion of multiple SOAs during the inhibitory period. The neural activation pattern instead supports more general attentional models that promote functional overlap for these networks (Salmi et al., 2009; Macaluso and Doricchi, 2013; Alho et al., 2015) rather than models which suggest that the networks differ (ventral: stimulus-driven; dorsal: goal-directed) in their specific attentional orienting functions (Corbetta and Shulman, 2011; Vossel et al., 2014). Importantly, these results may be linked to the modality of stimulus presentation, with previous fMRI studies suggesting that activation of the frontoparietal networks may be more similar for both top-down and bottom-up orienting in the auditory relative to the visual modality (Salmi et al., 2007, 2009; Alho et al., 2015) as well as between the auditory and visual modalities (Shomstein and Yantis, 2004; Yang and Mayer, 2014). However, to truly comprehend the extent of these modality activation differences in exogenous orienting, it is necessary to include both visual and auditory stimuli for direct comparisons.

There are several limitations associated with the current study that should be noted. The first is that pure-tone audiometry was not used to verify participants’ hearing threshold, however, we did require that participants were proficient in distinguishing cue and target tones before collecting data. Second, the range of the validity index was limited (i.e., closer to 0) with small effect sizes for all of three SOAs exhibiting behavioral evidence of IOR. This in turn may have limited our ability to observe weaker underlying relationships between RT and functional data in spite of the large sample size utilized in the current study. The current task could also have resulted in a priming of motor response (i.e., right cue – right middle finger), which may have subsequently influenced response time and functional data. In addition, the current study did not utilize a sparse sampling acquisition technique, which could have affected the pattern of functional activation due to the continuous ambient noise resulting from the switching of the gradient coils. A continuous, rather than sparse sampling, sequence was purposefully chosen for the current experiment to maximize the number of trials collected across four separate SOAs and to decrease the abrupt onset of scanner noise. The latter could have potentially triggered an additional orienting response that would have become confounded with task-related hemodynamics given the low temporal resolution of fMRI. Moreover, our behavioral data were similar to previous behavioral and electrophysiological studies that did not contain scanner noise, suggesting that the appropriate cognitive constructs were engaged during the task in spite of the null findings between the dorsal and ventral networks.

In summary, current results suggest a strong behavioral shift in exogenous auditory orienting from facilitation to inhibition across four different SOAs. Activation within the right VLPFC/anterior insula was highest during the facilitatory state, and activation within the right VLPFC (invalid – valid) further correlated with the degree of behavioral reorienting at the 200 ms SOA. Thus, current and previous results suggest that the VLPFC plays a role when auditory attention must be quickly deployed or redeployed following targets at short SOAs, requiring a heightened demand for cognitive and inhibitory control. Current results also indicated that the ventral and dorsal attention networks were activated for both valid and invalid trials regardless of whether an overall state of inhibition or facilitation was being evoked from a behavioral perspective. Thus, current results indicate that the dorsal and ventral networks may not be as specialized during bottom-up auditory attention as has been previously reported during visual orienting studies from a neural perspective. Future studies should consider the utilization of magnetoencephalography to further query the interaction between the dorsal and ventral frontoparietal networks during exogenous orienting. The increased temporal resolution could disambiguate networks responsible for the processing of auditory cues vs. those involved in the identification of the target.

## Ethics Statement

This study was carried out in accordance with the recommendations of the University of New Mexico (UNM) Health Sciences Center Human Research Protection Program (HRPP), UNM Human Research Review Committee with written informed consent from all subjects. All subjects gave written informed consent in accordance with the Declaration of Helsinki. The protocol was approved by the UNM Human Research Review Committee.

## Author Contributions

AM conceived of the experiment. FH, JL, JB, CA, and AM all had substantial input into the design of the experiment. FH, JL, AD, and AM all contributed to the collection, analysis, and interpretation of the data. All authors helped write the manuscript and approved of its final version.

## Conflict of Interest Statement

The authors declare that the research was conducted in the absence of any commercial or financial relationships that could be construed as a potential conflict of interest.
